# A First Step toward the Clinical Application of Landmark-Based Acoustic Analysis in Child Mandarin

**DOI:** 10.3390/children8020159

**Published:** 2021-02-20

**Authors:** Chin-Ting Liu

**Affiliations:** Department of Applied English, National Chin-Yi University of Technology, Taichung 411030, Taiwan; ctjimboliu@gmail.com

**Keywords:** acoustics, landmark analysis, Mandarin Chinese, language acquisition, consonant

## Abstract

As an initial step for the clinical application of landmark-based acoustic analysis in child Mandarin, the study quantified the developmental trajectories of consonants produced by four-to-seven-year-old children who acquired Taiwanese Mandarin as their first language. The results from a total of 80 children (20 in each age group, with gender balanced) indicated that younger age groups produced more +*b* landmark features than seven-year-olds did, showing that the development of obstruents was not completed by the age of six. A multiple regression showed that the participants’ speech intelligibility scores could be predicted by landmark features. Additionally, the +*b* landmark feature demonstrated the strongest net effect on speech intelligibility scores. The findings indicated that: (a) the landmark feature +*b* was an essential indicator of speech development in child Mandarin and; (b) the consonantal development in child Mandarin could be predicted by the physiological complexity of the articulatory gestures. Future studies focusing on a wider range of population (e.g., typically developing adults, aging and other clinical groups) with different language backgrounds are encouraged to apply landmark-based acoustic analysis to trace the linguistic development of a particular group.

## 1. Introduction

A massive body of literature has pointed out that the traditional manual segmentation and acoustical analyses of speech are too laborious and time-consuming (c.f. [1,2,3,4,5,6], among many others). As the transcription and coding are labor-intensive, the number of instances included in analyses is usually limited (c.f. [1,7]). This issue is particularly critical for pediatricians and language therapists because young children with high risks of speech disorders usually have limited energy and attention span. Therefore the speech evaluation sessions are less likely to be long enough to include a larger corpus of speech data produced by the children. In view of this limitation, several newly created devices and software have emerged with the aim of enabling researchers to analyze a larger body of samples with high validity and reliability without consuming too much time. SpeechMark© (Boston, MA, USA) [8] is one of those products and is built upon previous works by Liu [9] and Howitt [10].

SpeechMark© has been developed based on the landmark-based theory proposed by Stevens [11,12,13,14,15]. Unlike the traditionally proposed articulator-bound features (c.f. [16,17]), landmark-based analysis is an articulator-free analysis that focuses on the rapid change in spectrum or amplitude. These abrupt changes in spectrum or amplitude are said to correlate with speech intelligibility [18,19,20,21,22]. That is, listeners rely on those changes to judge what the perceived speech sounds are. At first, there were three types of landmarks, including ±*g* (lottis), ±*b* (urst) and ±*s* (onorant) (c.f. [9]), where the symbols ‘+’ (positive) and ‘−’ (negative) refer to the onset and the offset of the feature, respectively. Additional features are added when researchers develop SpeechMark© based on their observations of speech recordings. The specifications and the articulatory interpretations of the six abrupt-consonantal landmarks based on DiCicco and Patel [23], MacAuslan [24], Ishikawa, MacAuslan and Boyce [19], Atkins, Boyce, MacAuslan and Silbert [21], Huang, Epps and Joachim [25] and Ishikawa, Rao, MacAuslan and Boyce [22] are summarized in Table 1. Landmark-based acoustic analysis has been used to study the linguistic behaviors of several populations, including typically developing (TD) adults [18,19], individuals with dysarthria [23], children with cleft lip and palate [20], simultaneous bilingual children [26] and individuals with dysphonic speech [22].

For the following reasons, landmark-based acoustic analysis, by using SpeechMark©, would be particularly informative for researchers, pediatricians and language therapists to quantify the developmental trajectory of consonants produced by Mandarin-acquiring children. First, infants and young children’s productions are quasiphonetic [27] or are protophones [28,29]. That is, their productions might or might not have clear vowels and consonants and could not be sensibly transcribed with the symbols found in the International Phonetic Alphabet. Additionally, human listeners perceive sounds in the categorical fashion [30]. Therefore, categorizing and studying children’s productions with the articulator-bounded method might risk incorporating children’s protophones into adults’ existing sound inventories and failing to reliably represent those children’s productions. For instance, Zhu and Dood [31] and Zhu [32] study the consonantal acquisition of Mandarin-acquiring children by inviting human judges to transcribe those children’s word productions. The results indicate that the voiceless alveolo-palatal fricative/ɕ/and the voiceless alveolar fricative/s/are acquired sometime before those children are three years old and four and half years old, respectively. However, by studying the same segments with acoustical analyses, Li and Munson [33] show that the spectral energy distribution and the values of the second formant onset frequency of the following vowels generated by five-year-olds (the oldest age group in the study) are still different from those produced by adults. This shows that the adoption of acoustical methods in child language research is essential and could enable researchers to analyze and compare children’s speech without associating their quasiphonetic productions with a transcriber’s mental phonetic inventory.

Second, an objective and reliable reference of the consonant developmental trajectory in Mandarin Chinese provides a significant contribution in clinical settings. Although many studies have investigated how Mandarin-acquiring children acquired consonants, those studies set different correction rates, including 70, 75, and 90%, as the criteria for acquisition (c.f. [31,32,34,35,36,37,38,39,40,41,42,43,44,45]; and also [46] for a relevant literature review). That is, once the correction rate of a certain segment produced by a child is higher than the predetermined percentage (i.e., 70, 75, or 90% depending on independent studies), the acquisition of the segment is said to be completed. The differences in the criteria of acquisition results in the inconsistent order of consonantal acquisition reported in the literature. For instance, while some studies claim that the voiceless labiodental fricative /f/ is acquired later than the voiceless alveolar fricative /s/ [35,36], other studies claim that the segment /f/ is acquired earlier than the segment /s/ [31,32]. With the inconsistency of the order of the segmental acquisition, pediatricians and language therapists do not have a reliable and valid reference from the typical population when they assess the speech development of a potentially high-risk individual or when they wish to evaluate the progress in speech development of a particular atypical population. By using landmark-based acoustic analysis and the program SpeechMark©, the developmental trajectories of Mandarin consonants could be efficiently and reliably quantified, which in turn serve as essential references in clinical settings.

As the first step toward the clinical application of landmark-based acoustic analysis in child Mandarin, the purpose of the current study is to quantify the consonantal productions from four-to-seven-year-old Mandarin-acquiring TD children in Taiwan by using landmark-based acoustic analysis. Furthermore, the relationship between landmark features and speech intelligibility is explored. Children ranging from four to seven are selected because the literature shows that most consonants in Mandarin Chinese are acquired before five years old, with some fricatives and affricates being acquired after the age of six (c.f. [39,42,43,44,45]). The study is significant in the following aspects. First, based on the review in this section, it is clearly shown that an objective, efficient and reliable reference of the consonantal development of child Mandarin is in great need. The program SpeechMark©, based on the landmark-based theory proposed by Stevens [11,12,13,14,15], might be particularly informative in this respect. Second, the results could be used to test the prediction from the Biological Model of phonetic/phonological development proposed by Kent [27] and Locke [47]. Specifically, the Biological Model claims that the order of the segmental acquisition could be predicted based on the complexity of the speech motor control ability required in the articulation. According to the model, the articulatory gestures for producing fricatives and affricates require higher physiological demand in speech motor control ability. Therefore, those segments are said to be acquired sometime after children are six years old. Based on the model, it is predicted that the ±*b* and ±*f* features might be the informative indices, and the oldest age groups would demonstrate differences in these two features. Finally, the results of the study could shed light on the relationship between the landmark features and speech intelligibility.

## 2. Methods

### 2.1. Participants

Speech samples from 80 participants were included in the analysis. Table 2 summarizes the demographics of the participants. The author actively contacted and visited several kindergartens and elementary schools. After knowing the purpose, the methodology and the inclusion criteria for the participants, the chairpersons or class advisors of the institutions helped first screen the potential participants in the institutions. Specifically, the inclusion criteria required that all the participants acquired Taiwanese Mandarin as their first language and, according to the teachers and those children’s caretakers, did not have language-, learning- or hearing-related disorders. After that, the teachers at the institutions contacted the parent(s) or the caregiver of the potential participants. One of the parents of each participant was required to sign the consent form so that the experimenters could invite the child participants to join the recitation task individually at the kindergarten/elementary school they attended.

### 2.2. Equipment, Procedures and Materials

The unidirectional microphone RODE (NTG3B) was linked to the interface Babyface Pro, which was linked to the DELL Inspiron 15-5570 laptop. The same laptop was used to display the pictures used to elicit the participants’ productions. Praat [48] was the software used to record the speech productions from the participants. The sampling rate was set at 44.1 kHz. All the devices had been settled in a quiet room before the experiment formally started. As the unidirectional microphone was used, the ambient noise, if any, could be minimized or eliminated while recording.

A trained experimenter conducted all the recordings. When a child participant entered the quiet classroom in the kindergarten or the elementary school he/she attended, the experimenter invited the child to sit in front of the laptop. After that, the experimenter first verbally interacted with the child with the unidirectional microphone so that the child could be familiar with speaking to the microphone. When the experiment formally started, the experimenter invited the child participant to name the picture they saw. When the participants failed to produce the target word, the experimenter would recite the correct word and invited the child to repeat it. The microphone was held by the experimenter and he would constantly pay attention to the distance between the microphone and the participant’s lips. When children’s productions overlapped with noise (e.g., the bell ring at the elementary school), the experimenter would invite the child participant to reproduce those words again. After each participant completed the recitation task, he/she could choose three cartoon stickers as rewards.

Ten disyllabic words were included in the analysis and are listed in the Appendix A. The data were collected based on the two projects conducted by the author. As the contents and the length of the word lists used to elicit productions differ among different age groups, the 10 words that were shared among these age groups were included in the analysis.

### 2.3. Data Analysis

#### 2.3.1. Landmark-Based Acoustic Analysis

One trained assistant first screened the collected sound files and edited them so that the irrelevant sounds (e.g., the sounds from the experimenter and the disyllabic words that were not included in this study) could be deleted. The author double-checked the resulting edited sound files to make sure that all and only the 10 critical disyllabic words were included. After that, the same trained assistant ran the program SpeechMark© (WaveSurfer Plug-in, Windows Edition, Version 1.0.39) to generate the acoustic landmarks for each participant. The “infants” option was selected so that the range of fundamental frequency in the analysis was adjusted to the range from 1200 to 8000 Hz [24]. A custom-written program was used to automatically sum up the number of instances of each landmark symbol.

#### 2.3.2. Intelligibility Scores

A full-time licensed language therapist with more than 17 years of experience in practice was invited to provide the intelligibility score for each participant. The language therapist did not know the purpose of the study and the data presented to her were randomized. A 5-point Likert scale was adopted where a score from 1 to 5 represents that the speech productions were completely unintelligible (1), mostly unintelligible (2), somewhat intelligible (3), mostly intelligible (4), and completely intelligible (5), respectively. The language therapist gave a score to each disyllabic word production, and the final intelligibility score of each participant was the average score from his/her 10 productions.

#### 2.3.3. Statistical Analysis

Kruskal–Wallis H Test, the non-parametric equivalency of one-way ANOVA, was used to explore if there were differences in the total number of landmark features and within each landmark type among the four age groups. The total number of each landmark was the dependent variable, and the age was the independent variable. IBM SPSS Statistics Version 26.0 was the software used to run the statistical tests. Two notes are appropriate here. First, the landmark features whose total instances were fewer than 80 were not included for statistical analyses. As 80 participants were included in the current study, a landmark feature with a total number of instances less than 80 implies that on average each of the participants generated the feature less than once in the speech sample. In this case, the specific landmark feature was not sensitive enough to detect the speech signals produced by the participants and would not be able to inform us much about the developmental trends of the specific aspects of those children’s consonantal productions. Second, as this study is the first study to analyze Mandarin-acquiring children’s consonantal development by using the acoustic landmark analysis, increased risk of Type 1 errors was considered less of a concern than Type 2 errors. Therefore, the significant alpha value was set at 0.05. However, when there was a main effect for a specific landmark feature, six specific post hoc comparisons (age 7 vs. age 6, age 7 vs. age 5, age 7 vs. age 4, age 6 vs. age 5, age 6 vs. age 4, and age 5 vs. age 4) were computed to investigate if there were any differences in each landmark feature across different age groups by using Mann–Whitney U Test. In this case, the Bonferroni correction method was adopted, and the *p* value was set at 0.008 (i.e., 0.05/6).

A multiple regression was run using IBM SPSS Statistics Version 26.0 software to investigate how much of the variation in speech intelligibility scores could be explained by the landmark features. The dependent and independent variables were the individual participants’ speech intelligibility scores and the numbers of each landmark feature, respectively. As there was only one test for the regression analysis, the *p* value was set at 0.05.

## 3. Results

### 3.1. Descriptive Results

The results of the landmark-based acoustic analysis and the intelligibility scores were summarized in Table 3 and Table 4, respectively. According to the standard described in Section 2.3.3, four landmark features, including +*f*, −*f*, +*v*, and −*v*, were excluded from the later statistical analyses. In terms of the speech intelligibility scores, the four age groups demonstrated highly intelligible speech productions.

### 3.2. Inferential Results

Nine Kruskal–Wallis H Tests were performed to explore whether there were any differences in the number of landmark features (total landmarks without ±*f* & ±*v,* +*g,* −*g,* +*p,* −*p,* +*b,* −*b,* +*s,* and −*s*) among different age groups. The results showed that there was a statistically significant difference in the number of +*b* landmarks among different age groups, *χ^2^*(3) = 14.07, *p* = 0.003. No other comparisons were statistically significant. Six post hoc tests, using Mann–Whitney U Test, were performed to compare the number of +*b* landmarks produced by age 7 vs. age 6, age 7 vs. age 5, age 7 vs. age 4, age 6 vs. age 5, age 6 vs. age 4, and age 5 vs. age 4. The results indicated that the differences among three comparisons were statistically significant (age 7 vs. age 6: *U* = 73.5, *z* = −3.46, *p* = 0.001; age 7 vs. age 5: *U* = 103, *z* = −2.64, *p* = 0.008; age 7 vs. age 4: *U* = 92.5, *z* = −2.92, *p* = 0.003). In short, the results from the statistical analyses revealed that, except for +*b*, the differences in the numbers of the landmark features produced among 7-year-olds, 6-year-olds, 5-year-olds and 4-year-olds were not statistically significant. Seven-year-olds produced fewer +*b* acoustic landmarks than did other age groups.

A multiple regression analysis was performed in order to investigate how much of the variation in speech intelligibility scores could be explained by the landmark features. The results showed that these landmark features statistically significantly predicted speech intelligibility scores, *F* (8, 71) = 2.405, *p* = 0.023, *R^2^* = 0.213. That is, 21.3% of the total variation in speech intelligibility scores could be accounted for by all the eight landmark features (excluding +*f*, −*f*, +*v*, and −*v*). The landmark feature that added statistically significantly to the prediction was the +*b* feature (*p* = 0.0002, *B* = −0.031). For every one point increase in the number of the +*b* feature, speech intelligibility scores would be expected to decrease by 0.031 point.

## 4. Discussion

As the first step toward the clinical application of landmark-based acoustic analysis in child Mandarin, this study was designed to quantify the consonantal productions from Mandarin-acquiring children in Taiwan by using landmark-based acoustic analysis. Furthermore, the relationship between the landmark features and speech intelligibility scores was explored. The disyllabic word productions from 80 children (from four to seven years old) were collected and analyzed by using the program SpeechMark©. The results indicated that seven-year-olds produced statistically significantly fewer +*b* landmark features than did other age groups. No other statistically significant differences were found among these children’s productions. Additionally, all the participants hardly generated ±*f* and ±*v* landmark features. The results from a multiple regression analysis indicated that the eight landmark features (excluding +*f*, −*f*, +*v*, and −*v*) could statistically significantly account for 21.3% of the total variation in speech intelligibility scores. The net effect of the landmark feature +*b* was the strongest. For every one point increase in the number of the +*b* feature, speech intelligibility scores would be expected to decrease by 0.031 point. Based on the obtained results, several issues are discussed.

Three landmark features, +*b*, ±*f* and ±*v*, are first discussed below. First, the presence of the +*b* landmark represented the presence of bursts among obstruents [21,22]. The presence and absence of bursts had been consistently reported to be an essential indicator in speech intelligibility in both English and Chinese [49,50]. Empirical studies also demonstrated that TD young children produced more bursts for affricates than for fricatives [51]. That was an expected phenomenon as a release burst was expected for the first half stop in an affricate (e.g., the/t/in/ts/). Additionally, literature generally agreed that even the consonants with the more complicated articulatory gestures (e.g., the voiceless retroflex fricative/ʂ/) were virtually mastered around six years old [39,42,43,44,45]. Therefore, the fact that younger children (four to six years old) produced too many +*b* landmark features indicated that their finer-grained ability to properly control the speech motor was still developing and that progress was observed by the time children reached seven years old. Second, the scarcity of the two speech landmarks, ±*f* and ±*v*, deserves some attention. According to Huang, Epps and Joachim [25] and Ishikawa et al. [22], ±*f* is an indicator of the onset/offset of voiceless fricatives while ±*v* is an indicator of the onset/offset of voiced fricatives. As all the six fricatives in Taiwanese Mandarin (i.e., /x, ɕ, s, ʂ, ʐ, f/) are voiceless, the scarcity of the landmark feature ±*v* is understandable. At first glance, the scarcity of the landmark feature ±*f* might suggest that even the oldest children in the experiment might not be able to properly produce fricatives. However, a closer look at the acoustic rules in Table 1 and the existing literature might reveal a different picture. In fact, the ±*f* and ±*b* landmark features partially share acoustic rules (i.e., at least three of five frequency bands show simultaneous power increases/decreases of at least 6 dB). Nevertheless, ±*f* (and also ±*v*) further required the lower frequency bands to simultaneously decrease (or increase) when the higher frequency band showed power increases (or decreases). As the acoustic rules for ±*f* were more complicated and might be designed to detect a very rare case of fricatives, it was not surprising to learn that the ±*f* landmark features were scarce, if not totally unavailable, even among TD adult speakers in Chinese [52] and English [19,22]. These phenomena also indicated that the addition or modification of the landmarks that could be used to distinguish among stops, affricates and fricatives would be especially informative. The redefinition of the acoustic rules for detecting the landmark features ±*f* and ±*v* might be a solution. At first, the rules for detecting ±*f* and ±*v* must be less complicated so that fricatives in general could be detected. Second, as fricatives are the only obstruents that are produced without a stop burst, the acoustic rules for +*f* and +*v* should specify the timing of the detection. Specifically, the landmark features +*f* and +*v* could only be detected without a preceding +*b* feature within a certain time domain. In short, the +*b* landmark features, but not the ±*f* features, are more sensitive to the quality of the voiceless obstruents produced by Mandarin speakers. The redefinition of the acoustic rules for the landmark features ±*f* and ±*v* is required in order for the analysis to precisely determine the differences among different obstruents.

The current findings also lent strong support to the Biological Model of phonetic/phonological development proposed by Kent [27] and Locke [47]. According to the model, the phonetic/phonological development of children was substantially affected by their speech motor control ability. Based on Kent [27], consonants involving the fine force control to generate frication (i.e., fricatives and affricates) were acquired the latest, and the completion of the acquisition was sometime after children were six years old. The current findings matched the developmental trajectory predicted by the Biological Model. According to the experimental results, by age seven, Mandarin-acquiring children had made progress in the production of the +*b* landmark features (i.e., producing fewer +*b* landmark features). Please recall that the +*b* landmark features indicated the presence of a burst consonant. The higher number of +*b* landmark features among younger age groups implied that those children were more likely to generate stop bursts for segments even when such stop bursts were not expected (i.e., for fricatives). This phenomenon in turn showed that four-to-six-year-olds were less likely to properly generate the fine force regulation for frication and therefore produced the bursts that were not supposed to be present. In short, the current findings showed that the landmark feature +*b* is particularly sensitive to those children’s speech motor control ability.

A note about the relationship between landmark features and speech intelligibility is appropriate here. Boyce et al. [18] showed that speakers with clearer speech produced a higher number of landmark features. Similarly, Ishikawa, MacAuslan and Boyce [19] hypothesized that the greater number of landmark features produced by female speakers in their study might indicate greater intelligibility of their speech. However, according to the current findings from the landmark feature +*b*, the relationship between the number of landmark features and the degree of speech intelligibility might not always be “the more, the better”. According to the results from the multiple regression in the current study, the increase of the landmark feature +*b* resulted in the decrease in the speech intelligibility scores. Similar patterns were also reported in Ishikawa et al. [22]. In their study, Ishikawa et al. [22] compared the acoustic landmark features produced by dysphonic speakers and TD speakers. The findings indicated that the speakers from the clinical group produced a statistically significantly higher number of ±*g* and ±*b* features than did the control group. If the higher number of the landmark features indicated speakers’ better speech intelligibility, it was hard to justify why those individuals with dysphonic speech produced higher numbers of the landmark features ±*g* and ±*b*. Therefore, the findings from the literature and the current study suggest that, with regard to landmark features, “the more, the better” is inaccurate. Rather, it is more accurate to say that too many and too few acoustic landmark features would exert equally negative influences on speech intelligibility. As different languages encompass different segmental inventories, the critical landmark features that are strongly related to speech intelligibility might vary from language to language. Future studies focusing on languages other than Taiwanese Mandarin are suggested to directly explore the relationship between each of the acoustic landmark features and speech intelligibility so that researchers could identify the key landmark features that could account for the variation of speech intelligibility in the particular language.

The landmark-based acoustic analysis reported in the current study could be practically applied to several domains. First, as the landmark feature +*b* reflects the Mandarin-acquiring children’s speech motor control ability and exerts influences on speech intelligibility, future clinical applications of the analysis should focus on the quantity of the +*b* landmark feature that Mandarin-acquiring children with speech related disorders generate in their word productions. Second, it has been reported that aging people and elderly people with Parkinson’s disease generally have decreased speech motor control ability and lower speech intelligibility. In this case, for Mandarin-speaking adults, it is expected that seniors would produce more +*b* landmark features than did the younger generations. In addition, those Mandarin-speaking individuals with Parkinson’s disease might also produce more +*b* landmark features than did their TD counterparts. In short, the application of landmark-based acoustic analysis to various TD or disordered populations would inform us about the nature of those individuals’ speech motor control ability.

## 5. Conclusions

By using landmark-based acoustic analysis, the current study quantified the consonantal developments among children ranging in age from four to seven years. The results of the disyllabic word recitation task indicated that the younger children (four, five and six-year-olds) produced a significantly higher number of the +*b* landmark features than did the seven-year-olds. In addition, the number of the +*b* landmark features were negatively correlated with the participant’s speech intelligibility scores. The experimental results could be elegantly accounted for by the Biological Model of children’s phonetic/phonological development [27,47], which claimed that consonants requiring finer-grained speech motor control ability were acquired sometime after the age of six. Additionally, based on the literature and the current study, it could be concluded that the relationship between the number of landmark features and speech intelligibility is not always “the more, the better”. Instead, too many and too few acoustic landmark features would exert equally negative influences on speech intelligibility. Additionally, the acoustical rules for detecting the landmark features ±*f* and ±*v* should be refined so that the distinctions among obstruents could be more precisely identified. Pediatricians and language therapists are encouraged to apply landmark-based acoustic analysis in clinical sessions, and the findings from the TD children presented in the current study could serve as essential references for the Mandarin-acquiring population.

## Figures and Tables

**Table 1:** Acoustic rules and articulatory interpretations of the six abrupt-consonantal landmarks.

Symbol	Mnemonic	Acoustic Rule ^1^	Articulatory Interpretation
±*g*	Glottal	Beginning/end of sustained laryngeal vibration/motion	Onset/offset of vocal folds’ free vibration
±*p*	Periodicity	Beginning/end of sustained periodicity (syllabicity) lasting for at least 32 milliseconds	The presence of ±*p* reflects the speaker’s ability to properly control the subglottal pressure and cricothyroid muscle.
±*b*	Burst	At least three of five frequency bands show simultaneous power increases/decreases of at least 6 dB in both the finely smoothed and the coarsely smoothed contours in an unvoiced segment (not between +*g* and the next −*g*)	Presence of a fricative, affricate or aspirated stop burst consonant (i.e., +*b*) or cessation of frication or aspiration noise (i.e., −*b*)
±*s*	Syllabic	At least three of five frequency bands show simultaneous power increases/decreases of at least 6 dB in both the finely smoothed and the coarsely smoothed contours in a voiced segment (between +*g* and the next −*g*)	Closure or release of a nasal or /l/
±*f*	Unvoiced frication	At least three of five frequency bands show simultaneous 6 dB power increases/decreases at high frequencies and decreases/increases at low frequencies (unvoiced segment)	Onset/offset of an unvoiced fricative
±*v*	Voiced frication	At least three of five frequency bands show simultaneous 6 dB power increases/decreases at high frequencies and decreases/increases at low frequencies (voiced segment)	Onset/offset of a voiced fricative

^1^ The descriptions of the rules are from [24].

**Table 2:** Demographics of the participants in the current study.

Participants	Total Participants (Number of Girls)	Mean Age in Month (*SD*) ^1^
4-year-olds	20 (10)	52.25 (2.552)
5-year-olds	20 (10)	64.20 (2.821)
6-year-olds	20 (10)	76.20 (2.353)
7-year-olds	20 (10)	88.20 (2.238)

^1^*SD* stands for standard deviation.

**Table 3:** Mean and standard deviation (in parentheses) of the landmark features produced by each age group.

Landmark Features	Age 4 (*n* = 20)	Age 5 (*n* = 20)	Age 6 (*n* = 20)	Age 7 (*n* = 20)
+*g*	19.25 (2.79)	18.55 (2.54)	18.4 (1.7)	18.9 (3.49)
−*g*	19.25 (2.79)	18.5 (2.48)	18.45 (1.73)	18.85 (3.5)
+*p*	26.9 (7.82)	23.95 (4.71)	24.8 (5.03)	25.55 (6.33)
−*p*	24.6 (5.753)	22.35 (3.56)	23.3 (4.14)	24 (5.54)
+*b*	10.05 (3.33)	9.75 (3.73)	10.5 (3.17)	7 (3.1)
−*b*	3.45 (2.31)	2.75 (1.68)	2.8 (1.58)	2.8 (2.09)
+*s*	5.55 (3.09)	5.2 (3.3)	4.7 (3.23)	4.95 (3.4)
−*s*	5.3 (2.96)	5.55 (3.1)	5.4 (2.8)	4.6 (4.31)
+*f*	0.15 (0.49)	0 (0)	0 (0)	0 (0)
−*f*	0.1 (0.31)	0.05 (0.224)	0.15 (0.366)	0 (0)
+*v*	0.05 (0.22)	0 (0)	0.1 (0.31)	0.05 (0.22)
−*v*	0.45 (0.83)	0.25 (0.55)	0.05 (0.22)	0.15 (0.366)
Total	115.1 (17.47)	106.9 (11.35)	108.65 (12.87)	106.85 (16.6)
Total without ±*f* & ±*v*	114.35 (17.37)	106.6 (11.39)	108.35 (12.93)	106.65 (16.69)

**Table 4:** Mean and standard deviation (in parentheses) of the intelligibility scores produced by each age group.

Age 4 (*n* = 20)	Age 5 (*n* = 20)	Age 6 (*n* = 20)	Age 7 (*n* = 20)
4.825 (0.259)	4.905 (0.267)	4.855 (0.305)	4.975 (0.079)

## Data Availability

The data presented in this study are available on request from the author. The data are not publicly available due to original informed consent provisions.

## Appendix A

The 10 disyllabic words that were analyzed in the study. Please note that the participants saw pictures of the object and did not read the contents below.

**Table A1:** Ten disyllabic words included in the present study.

No.	Chinese Characters	Transliteration (Pinyin)	Gloss
1.	鳳梨	fèng lí	Pineapple
2.	飛機	fēi jī	Airplane
3.	火車	huǒ chē	Train
4.	漢堡	hàn bǎo	Hamburger
5.	蝦子	xiā zi	Shrimp
6.	小鳥	xiǎo niǎo	Bird
7.	森林	sēn lín	forest
8.	松鼠	sōng shǔ	squirrel
9.	薯條	shǔ tiáo	french fries
10.	手錶	shǒu biǎo	watch
